# Link between insulin resistance and hypertension: What is the evidence from evolutionary biology?

**DOI:** 10.1186/1758-5996-6-12

**Published:** 2014-01-31

**Authors:** Ming-Sheng Zhou, Aimei Wang, Hong Yu

**Affiliations:** 1 Department of Physiology, Liaoning Medical University, No. 40, Section 3 Songpo Road, Jinzhou, Liaoning, China; 2 Department of Cardiology, 2nd Affiliated Hospital, School of Medicine, Zhejiang University, Hangzhou, Zhejiang, China

**Keywords:** Insulin resistance, Hypertension, Evolution, Inflammation and sodium

## Abstract

Insulin resistance and hypertension are considered as prototypical “diseases of civilization” that are manifested in the modern environment as plentiful food and sedentary life. The human propensity for insulin resistance and hypertension is a product, at least in part, of our evolutionary history. Adaptation to ancient lifestyle characterized by a low sodium, low-calorie food supply and physical stress to injury response has driven our evolution to shape and preserve a thrifty genotype, which is favorite with energy-saving and sodium conservation. As our civilization evolved, a sedentary lifestyle and sodium- and energy-rich diet, the thrifty genotype is no longer advantageous, and may be maladaptive to disease phenotype, such as hypertension, obesity and insulin resistance syndrome. This article reviews human evolution and the impact of the modern environment on hypertension and insulin resistance.

## Background

Diminished tissue sensitivity to insulin is a characteristic of various pathological conditions termed the insulin resistance syndrome, also known as the metabolic syndrome or cardiometabolic syndrome [1]. The metabolic syndrome is not a single disease, but rather a complex cluster of symptoms that include a large waist circumference, hypertension, hyperglycermia, dyslipidemia and insulin resistance, all of which are commonly associated with increased risk of obesity and Type 2 Diabetes [2]. Since patients with metabolic syndrome are commonly afflicted with cardiovascular morbidities, the metabolic syndrome and cardiovascular diseases share common pathways including increased oxidative stress, defective glucose, lipid metabolism, low grade inflammation, hypercoagulability and endothelial damage. Previously, investigators proposed to use the “circulatory syndrome” to refine the metabolic syndrome concept through the addition of markers of cardiovascular diseases such as renal impairment, microalbuminuria, arterial stiffness and left ventricular dysfunction [2]. It has become increasingly obvious that insulin resistance and the efforts made by the insulin-targeted organs to compensate for this defect play a vital role in the pathogenesis and clinical course of the metabolic syndrome [3].

Insulin resistance and hypertension are the components of metabolic syndrome and often coexist [4]. Clinical studies have shown that about 50% of hypertensive individuals have hyperinsulinemia or glucose intolerance, whereas up to 80% of patients with type 2 diabetes have hypertension [4,5]. In addition to its metabolic effects, insulin induces vasorelaxation by stimulating the production of nitric oxide (NO) in endothelium [6] and regulates sodium homeostasis by enhancing sodium reabsorption in the kidney [7,8]; thereby, contributing to the regulation of blood pressure. Recent studies have demonstrated that insulin resistance can develop not only in the classic insulin-responsive tissues, but also in cardiovascular tissues where insulin participates in the development of cardiovascular diseases and hypertension [1,9]. Insulin resistance has gained a bad name and is perceived as deleterious: commonly associated with the metabolic syndrome and hypertension that confer an increased risk for type 2 diabetes and cardiovascular diseases [9]. However, in human evolutionary history, insulin resistance may be an essential part of normal homeostasis to facilitate redirection of nutrients to pivotal organs and a physiological adaptive mechanism to promote our ancestor’s survival in times of critical conditions; such as, famine, infection, trauma and stress [10,11]. The same mechanism may be inappropriately activated on a chronic basis on the current obesogenic environment, leading to the manifestation of hypertension, insulin resistance or metabolic syndrome [12]. This article reviews human evolution and the impact of modern environment on hypertension and insulin resistance.

### Insulin resistance and elevation of blood pressure as an adaptive mechanism to promote human survival

Human survival has relied upon the ability to withstand starvation through energy storage, the capacity to fight off infection by an immune response, and the ability to cope with physical stresses by an adaptive stress response [11]. The physiological adaptation that is induced by the fasting state includes increased lipolysis, lipid oxidation, ketone body synthesis, endogenous glucose production and uptake and decreased glucose oxidation [13]. These processes are crucial for survival and serve to protect the organism from excessive loss of protein mass. Humans are extremely sensitive to glucose deficits, due to the large energy requirement (glucose) of the brain [11]. The requirement for energy storage is essentially served by the anabolic actions of insulin, during starvation or infection/inflammation it becomes insulin resistant, along with many other adaptations [12]. The way to maintain glucose levels during starvation, pregnancy and infection/inflammation is through insulin resistance in insulin-dependent tissues [12,14].

Insulin is an anabolic hormone that plays an important role in the regulation of glucose, lipid homeostasis and energy storage through its metabolic effects on classic insulin-responsive tissues [1]. Specifically, insulin promotes the storage of glucose as glycogen in liver and skeletal muscles, and facilitates deposition of fatty acids in the form of triglycerides in adipose tissue [13]. During insulin resistance, insulin-mediated anabolic metabolic effects are inhibited in the classic insulin-responsive tissues. For example, adipose tissue and skeletal muscle reduce the uptake of glucose and storage of glucose as glycogen and triglycerides. Concommintently, there is an increase in the hydrolysis of stored triglycerides and their mobilization as free fatty acids and glycerol, in which the liver increases glucose production via gluconeogenesis and inhibition of glycogen synthesis and storage. Insulin resistance promotes reallocation of energy-rich substrates (glucose to the brain, fetus and immune system; fat to the fetus and the organs) and the compensatory hyperinsulinemia [13]. Therefore, negative regulation of insulin signaling could be viewed as a physiologic ‘adaptive mechanism” that is activated in certain conditions such as fasting, inflammation, stress and pregnancy. However, its persistence at a chronic state is the basis of the ultimate changes that we recognize as the symptoms of the metabolic syndrome [11].

Insulin has complex vascular actions that appear as either vascular protective or deleterious effects [1]. Vascular protective effects of insulin, including induction of vasorelaxation, inhibition of vascular smooth muscle cell (VSMC) proliferation and anti-inflammation, are mediated by stimulating nitric oxide-dependent (NO) mechanisms in the endothelium [1]. Vascular deleterious effects of insulin include induction of vasoconstriction, VSMC proliferation and proinflammatory activity. These vascular effects are mediated through the mitogen-activated protein kinase (MAPK) pathway [1]. In addition, insulin increases sodium reabsorption in the kidney and promotes sympathetic nerve activity [8]. Insulin can be both inflammatory and anti-inflammatory [1,15], in physiological condition insulin stimulates endothelial NO production to exert a vasorelaxation and anti-inflammatory effect. Whereas, in the state of insulin resistance, the insulin-stimulated NO pathway is selectively impaired and the compensatory hyperinsulinemia may activate MAPK pathway, resulting in enhancement of vasoconstriction, proinflammation, increased sodium and water retention and the elevation of blood pressure [15,16]. The increased blood pressure by insulin resistance may contribute to increased blood perfusion to the brain during starvation and infection, and to the fetus during pregnancy.

### Insulin resistance and hypertension are associated with an unhealthy lifestyle and a systemic low grade inflammation

Insulin resistance and hypertension are considered to be Western diseases. It has become clear that most, if not all, typically Western chronic diseases find their primary causes in unhealthy lifestyles and that systemic low grade inflammation is a common denominator [11,17,18]. Our ancestors (primitive humans) had to undertake considerable physical activity to gain food and had to adapt to prolonged period of famine [17], which favored fat storage, a trail inherited by modern man. Moreover, our modern lifestyle, characterized by energy- and sodium-rich Western diet, sedentary life and high psychosocial stress, favors positive energy balance. In the long term, this positive energy balance creates the need for surplus fat storage [18]. When the capacity for safe lipid storage in adipose tissue is exceeded lipids overflow to non-adipose tissue, increasing the risk for chronic systemic low grade inflammation and subsequent insulin resistance, hypertension and metabolic syndrome [19].

Obesity in humans may be considered as a symptom of energy imbalance: caloric intake exceeds energy expenditure [11]. The human organism has extensive fuel reserves in the adipose tissue, which may meet energy demands for substantial periods. The adipocyte is thought to be both a static storage depot for calories as triglycerides and an endocrine organ to secret many hormonal factors, including lipid mediators, stress kinases and proinflammatory cytokines and chemokines [11]. These molecules participate in regulating energy metabolism, lipid storage, and inflammatory responses. In addition, excess nutrient intake can induce oxidative stress in the adipose tissue [20]. Oxidative stress conversely exerts significant effects on adipose tissue biology and can lead to dysregulation of adipocyte function, which manifests as inhibited adipocyte differentiation, enhanced immune cell infiltration into adipocytes and increased inflammatory cytokine secretion [21].

Obesity-induced inflammation is associated with increased adipose tissue macrophage (ATM) infiltration [22,23]. Similar to a pathogenic response to an invading bacterium, excess nutrients found in the obese adipose microenvironment can lead to the pro-inflammatory activation and phenotypic switch (from M2 resident to M1 inflammatory macrophage) of macrophage [24]. One of emerging feature of obesity-associated ATM infiltration is the linkage of the induction of a chronic inflammation in white adipose tissue (WAT) that eventually becomes systemic [22]. Excess WAT is an overactive endocrine organ secreting an array of inflammatory adipokines, such as tumor necrosis factor (TNFα), monocyte attractant protein 1 and interleukin 6(IL6) [24]. These inflammatory cytokines can not only induce a chronic inflammatory process in adipocyte tissue, but also be released into circulatory blood, inhibiting insulin signaling; resulting in global insulin resistance [25]. Therefore, chronic inflammation in obesity plays a critical role in pathogenesis of insulin resistance [26]. Ironically, the formation of a systemic and/or local tissue-specific insulin resistance due to inflammatory cell activation may actually be a protective mechanisms that co-evolved with the repartition of energy sources within the body during times of stress and infection [12].

Hypertension is also associated with increased systemic and vascular inflammatory responses and oxidative stress, which may contribute to vascular dysfunction [4,16]. Although the genetic causes of essential hypertension remain elusive, studies in Dahl salt-sensitive (DS) rat, a paradigm of salt-sensitive hypertension in human, have suggested that chromosome 2 contains quantitative trait loci for blood pressure and genes encoding inflammatory mediators with biological effects on T lymphocytes [27]. DS rats exhibit elevation of blood pressure, vascular inflammation, oxidative stress and endothelial dysfunction. These symptoms are reduced in the SSBN2 rat, a consomic rat, in which chromosome 2 of the DS rat is replaced by that of the normotensive Brown Norway rat [27]. Studies [16,28] in DS rats have shown that oxidative stress and activated oxdative stress-associated inflammation are linked not only to elevation of blood pressure and vascular dysfunction but also to insulin resistance, Moreover, antioxidant treatment and inhibition of the nuclear factor *κ*B inflammatory pathway in DS rats reduced blood pressure, vascular inflammation, and improved endothelial function, as well as, systemic and vascular insulin resistance. These studies support the notion that inflammation is a link between hypertension and insulin resistance [26].

### Link between insulin resistance and hypertension: what is the evidence from evolutionary biology?

Evolution by natural selection is a central organizing concept in biology. For millions of years, living creatures from lower-level organisms to human beings have been faced with survival stresses, including famine and infection [11]. The survival of multicellular organisms depends on the organism’s ability to store energy for times of low nutrient availability or high energy needs, and the ability to fight infections [29]. To meet the challenges of infection and other environmental stress, an activated immune system has an urgent need for energy-rich substrates that must be allocated from internal and external energy stores (glycogen, proteins, triglycerides, or free fatty acids) [30]. An activated immune system usually requires substantial energy in a quiescent state. This requirement rises into an active phase of inflammation [30]. Therefore, the metabolic and immune systems are among the most basic requirements across the animal kingdom [19,31]. It is not surprising then that metabolic and immune pathways have evolved to be closely linked and interdependent and that the genes controlling metabolic and pathogen-sensing systems have been highly conserved from lower-level organisms to mammals [32]. In recent years, new insights have been gained through multiple interactions between metabolic and immune system [19,32]. An increasing body of evidence suggests that energy metabolism is crucial for the maintenance of chronic inflammation, not only in terms of energy supply, but also in the control of the immune response through metabolic signals [11,33]. It is now apparent that critical proteins are necessary for regulating energy metabolism, such as peroxisome proliferator-activated receptors (PPAR γ), Toll-like receptors, and fatty acid-binding proteins. These critical proteins also act as links between nutrients metabolism and inflammatory pathway activation in immune cells [34,35]. For example, PPAR-γ a master regulator of adipocyte differentiation, is also a major molecule that drives the accumulation and phenotype of T^reg^ cells in adipose tissue [34]; and leptin, an important adipocyte-derived hormone to regulate energy homeostasis, can affect thymic homeostasis and the secretion of acute-phase reactants such as IL-1 and TNF α [36].

Under normal conditions, the integration of the metabolic and immune systems is fundamental for the maintenance of good health. The basic inflammatory response favors a catabolic state and inhibits anabolic pathways, such as the highly conserved insulin signaling pathway, resulting in insulin resistance [37]. As a result of insulin resistance, plasma levels of glucose are elevated to provide energy sources, maintain the function of vital organs, such as, the heart, brain, and immune cells, and combat infection. As the heart, brain and leukocytes are considered as insulin insensitive tissues [14], their energy metabolism is acutely dependent on plasma levels of glucose [18]. Therefore, insulin resistance resulting from acute inflammatory episodes may favor nutrient poor organisms to fight against infection. However, since most aspects in a living body occur with constraints on energy availability, regulation of energy storage and provisions occupy a very high position in the hierarchy of homeosotatic neuroendocrine immune control [38]. Energy regulation operates not only in the cell, but also in coordinating centers of the brain, and in endocrine organs that integrate organismal functions [37].

The time frame of an organism’s response to the acute inflammatory episode may reflect an adaptive natural selection mechanism for the coordination of the immune and energy system to fight against infection [39,40]. An acute infectious disease can be self-limiting, and may involve an innate immune response of 2-3 days; the subsequent phase of the adaptive immune response can last approximately 3 to 4 weeks [40]. Not only does the infection-induced impairment in health and the related anorexia exacerbate a significant reduction in intake of energy-rich substrates but an acute infectious disease can also be very energy consuming. Therefore, organisms must obtain the fuel from energy storage tissues, which primarily occur in fat tissue and skeletal muscle [11,41]. However, an increase of inflammatory cytokines released from infectious tissue into circulatory blood may result in fat and muscle insulin resistance to reduce energy consumption in these tissues, Additionally, a compensated hyperinsulinemia, high glucose and hyperlipidemia favor the body against infection [11]. While the energy from muscle and fat tissues usually last 3-5 weeks, perfectly matching an adaptive immune response to combat the infection [42]. Unfortunately, if an adaptive immune system cannot appropriately react within this time frame, the affected individual may die from energy exhausted [30].

With the exception of an energy requirement, acute inflammation is often accompanied by local and systemic water loss. Water loss includes local water loss from the exposed surface area of inflamed tissue as well as systemic water loss from insensible perspiration through skin and respiratory tract, and more metabolic water for higher metabolic reaction [40]. To overcome a systemic water loss during acute inflammatory episodes, a water retention system must be activated [40]. The reactive mechanism for water retention includes activation of sympathetic nervous system which subsequently results in activation of renin-angiotensin-aldosterone system, and activation of hypothalamic-pituitary-adrenal (HPA) axis with adrenocorticotropic hormone (ACTH), aldosterone and cortisol [40]. Interestingly, some hormones that mediate water retention such as angiotensin II and aldosterone are also endowed with proinflammatory effects [43], and has an important role in the pathogenesis of hypertensive and metabolic diseases [9].

The water retention system shows important similarities with the energy provision system. The operation of these two interacting systems were likely subject to positive selection and co-evolved to overcome serious and transient inflammatory episodes [40]. Induction of energy storage system and water retention system provides a survival mechanism in response to acute inflammatory episodes. However, prolonged operation of these adaptive programs such as in chronic inflammatory process, which are currently observed in many cardiovascular, hypertensive and metabolic diseases, can become pathogenic because there is no program to counteract continuous water retention and energy appeal actions [10,11].

### Is sodium another link between hypertension and insulin resistance?

Essential hypertension can be classified as salt-sensitive and salt-resistant, according to the blood pressure response to salt loading. Animal and clinical studies suggest that insulin resistance and hypertension are associated with salt-sensitivity [44,45]. High salt diet impairs insulin sensitivity in hypertensive patients with salt-sensitivity but not in those with salt-resistance [5]. There is a strong clustering of markers of endothelial damage in persons predisposed to salt-sensitive hypertension who concomitantly have insulin resistance and microalbuminuria [45].

Salt sensitivity of blood pressure is an independent risk factor for development of cardiovascular morbidity and mortality [5]. The essential hypertensive patients with salt sensitivity are more insulin resistant than those with salt-resistance. Furthermore, high salt diet impairs insulin sensitivity only in hypertensive patients with salt-sensitivity but not in those with salt-resistance, suggesting that there is a pathogenetic link among hypertension, salt-sensitivity and insulin resistance [4,28]. A recent clinical study has shown that insulin resistance enhances the blood pressure response to sodium intake^21^. Therefore, reduction in sodium intake may be an especially important component in reducing blood pressure in patients with multiple risk factors for insulin resistance and the metabolic syndrome [46].

The human propensity for hypertension is a product, at least in part, of our evolutionary history [47]. The evolution of hypertension susceptibility has been hypothesized to begin in Africa. With the hot and humid climate, effective heat dissipation is essential in hot environments and is achieved most efficiently through evaporative heat loss [47]. However, sweating due to the hot climate and excessive labor activities can lead to a large loss in the amount of salt and water, and eventually lead to hypovolemia, a threat to human survival. In addition, human and nonhuman primates living in ancient times had very low salt intake available. Low salt intake and large salt losses due to sweating had created robust salt appetite and renal sodium conservation, which were essential to survival. The principle of natural selection may have allowed the ancestral sodium-conserving genotype (thrifty) to persist [48], which may be maladaptive to the modern environment of sodium abundance, resulting in hypertension. An analogous evolutionary framework, sometimes referred to as the sodium-retention hypothesis, was proposed to explain the increased prevalence of essential hypertension in some ethnic groups. Briefly, ancient human populations living in hot, humid areas adapted to the environment by retaining salt. Whereas populations in cooler, temperate climates adapted to conditions of higher sodium levels [47,48]. Experimental evidence from trials of dietary sodium restriction generally supports the hypothesis of a sodium-hypertension link, particularly among salt-sensitive populations [49]. In support of this hypothesis, a study recently found that African Americans have a higher prevalence of salt sensitivity than White Americans [50].

Evidence suggests that genetic susceptibility to hypertension is ancestral [47]. Ancestral alleles increase the risk of hypertension and the derived protective alleles appear to carry the signature of positive selection at tightly linked neutral sites. For example, chimpanzees and humans share hypertension susceptibility alleles in at least two genes: angiotensinogen (AGT) and the epithelial sodium channel γ subunit (ENaCγ), genes involved in the regulation of sodium and blood pressure homeostasis [39]. AGT carries two variants: a promoter A-6G variant and the T235M variant, which are associated with hypertension [40]. Human genomic studies suggest that the genetic origins of susceptibility to common chronic disease are due to adaptations to ancestral environments [47], these alleles improved survival in ancestral environments characterized by salt scarcity, low-calories diets, and regular physical activity [39]. In the current environment of salt and caloric excesses and infrequent physical activity, these alleles can be detrimental, leading to obesity, type 2 diabetes and hypertension [48].

In salt-sensitive hypertension, the accumulation of sodium in tissue has been presumed to be accompanied by a commensurate retention of water to maintain the isotonicity of body fluids. Recent studies [51,52] suggest that immune cells, such as mononuclear phagocyte system and macrophages, are responsible for interstitial hypertonic sodium retention, resulting from high salt diet intake, and stimulate lymphcapillary network formation via production/release of tonicity-responsive enhancer binding protein and vascular endothelial growth factor C. Notably, vascular endothelial growth factor C may serve as an extra sodium and water storage in skin to buffer extracellular volume expansion and maintain blood pressure homeostasis [51]. Deletion of the mononuclear phagocyte system or inhibiting the interaction of VEGF-C with its receptors (VEGF receptor 3 and/or VEGF receptor 2) blocked the regulatory response of mononuclear phagocyte system to interstitial sodium accumulation and augmented salt-induced hypertension, suggesting that immune system plays the role in the regulation of sodium and water homeostasis.

Interestingly, insulin has been shown to inhibit sodium excretion by increasing sodium reabsorption in the kidney [8]. It is well known that sodium is the main determinant of body fluid distribution. Sodium accumulation causes water retention and often, high blood pressure. Sodium transport through various nephron segments, is quite important in regulating sodium reabsorption and blood pressure [8]. ENaC and sodium proton exchanger type 3 (NHE3) are main mediators to regulate sodium reabsorption in renal tubules. It has been shown that insulin can regulate ENaC and NHE3, therefore increasing renal tubular sodium reabsorption [8,53]. As mentioned earlier, like energy storage, insulin-mediated sodium preservation may be an adoptive mechanism for human survival during ancient time [7,54].

### Thrifty hypothesis

Natural selection shapes organisms in functioning within a particular set of environmental conditions [55]. Because organisms adapt to the totality of their environment, or ecological niche, it is hypothetically possible that natural selection favors organisms harboring the genotype for a metabolic system (such as the insulin signaling pathway) that has an increased response to inflammation [48]. Since regulation of energy storage and metabolism and the preservation of body fluids are critical for organism’s fight against famine, infection and physical stress, it has also been hypothesized that genes responsible for energy regulation and sodium preservation have been positively selected [48]. These genes were termed thrifty genes. The notion of thrifty genotype was initially proposed by Neel [56], in which he argued that certain genotypes were selected into the human genome because of their selective advantage over the less thrifty genes. Neel [56] defined a thrifty genotype as “being exceptionally efficient in the intake and/or utilization of food”. In ancient time, food supply was never consistent. Thus, it is contended that the ancient hunter-gatherer had cycles of feast and famine, punctuated with obligate periods of famine, and certain genes evolved to regulate efficient intake and utilization of fuel stores [14,56]. Subsequently, during famines, individuals with the thrifty genotype would have a survival advantage because they relied on larger, previously stored energy to maintain homeostasis [14]. Based on Neel’s thrifty genotype hypothesis, it is proposed that a genetic predisposition in developing diabetes was adaptive to the feast and famine cycles of Paleolithic human existence, allowing humans to fatten rapidly and profoundly during times of feast so that they may have a higher chance of survival during times of famine [55,56]. This would have been advantageous back then, but not in our current environment, as the current environment provides ready abundant energy rich food. Thus, the preserved thrifty genes that co-regulate energy storage and immune system may actually promote the development of obesity or type 2 diabetic mellitus [55].

Neel’s thrifty gene hypothesis has been challenged many times. One of the most significant problems for the thrifty gene hypothesis is that it predicts that modern hunter-gatherers should get fat in the periods between famines, but data on the body mass index of modern hunter-gatherer does not support this [57]. An alternative hypothesis, called the thrifty phenotype hypothesis, has been proposed. Thrifty phenotype hypothesis emphasizes life-course plasticity in the aetiology of variability in body composition and metabolism, and thrifty factors arising from a direct result of the environment within the womb during development [58]. The development of insulin resistance is theorized to be directly related to the body “predicting” a life starvation for developing fetus [58]. Another theory, thrifty epigenomic hypothesis, argues that an individual’s risk for metabolic diseases is primarily determined by epigenetic events, epigenetic modifications at many genomic loci alter the shape of thrifty genotype in response to environmental influences and thereby establish a predisposition for metabolic syndrome [59].

The concept of thrift has been widely associated with adiposity [14]. Recent studies emphasize that adiposity, like stature, is a polygenic trait, and that population genetic variability primarily comprises different frequencies of particular alleles, rather than major systematic differences [14,60]. Ethnic differences in body composition, representing different load-capacity ratios, may contribute to ethnic variability in metabolic risk. Lower lean mass and greater adiposity each indicate thrift. It has been proposed that body composition phenotypes, including the fat-lean ratio, the organ-muscle ratio, the central –peripheral ratio and the expandability of adipose tissue, are relevant to variability in the metabolic syndrome [60].

## Conclusions

**Figure 1:**
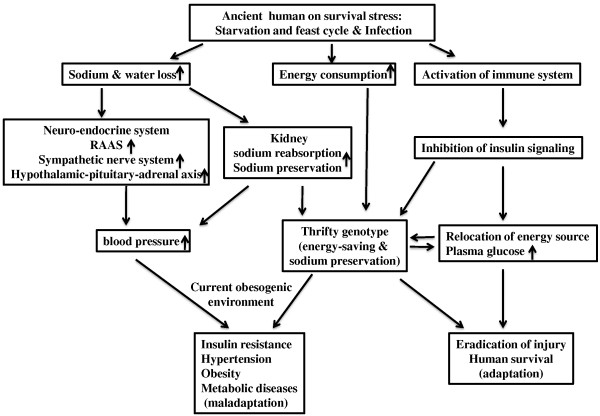
**A depiction of how natural selection of thrifty genotype**, **which was a physiological adaptive mechanism for human survival**, **on the current obesogenic environment, is maladaptive to disease phenotype.** Our ancestors were often faced with survival stresses, including famine, infection, trauma and physical stress. For example, an acute inflammatory episode may cause water loss, high energy consumption and activation of the innate and adaptive immune system. To cope with the injury responses, an elegant coordination of neuroendocrine, energy storage and immune systems are adapted. Inflammatory cytokines released from activated immune cells inhibits insulin signaling pathway; as a result, plasma levels of glucose are elevated to provide energy sources to maintain the function of vital organs (heart, brain and immune cells) and combat for the infection. In addition, water loss and sodium deprivation due to insufficient sodium intake or excess sodium loss may activate rennin-angiotensin-aldosterone system (RAAS), sympathetic nerve or neuro-endocrine system to preserve sodium and body fluid and increase blood pressure. As results of natural selection, the survival pressures drove our evolution to shape a thrifty genotype, which favored/promoted energy-saving and sodium preservation. With the switch to a sedentary lifestyle and sodium- and energy-rich diets (current obesogenic environment), the thrifty genotype is no longer advantageous, and may be maladaptive to disease phenotype, resulting in hypertension, obesity and insulin resistance syndrome.

Abundant clinical and epidemiologic evidences demonstrate a close linkage between insulin resistance and hypertension. The coexistence of insulin resistance and hypertension results in a substantial increase in the risk of developing cardiovascular disease and type II diabetes [5]. Underlying the mechanisms is complex and may involve a low grade chronic inflammation and oxidative stress. As humans evolve, the thrifty genotype for high cytokine responder (eradication of injury), mild insulin resistance (protection against starvation), or sodium preservation (maintenance of body fluid),which favored our ancestors, aiding them in the survival of critical conditions such as famine, infection, trauma and physical stressors, may be positively selected, which may be maladaptive to our current, modern lifestyle, resulting in insulin resistance, hypertension, type II diabetes and cardiovascular diseases (Figure 1) [12,61].

## Competing interests

The authors declare that they have no competing interests.

## Authors’ contributions

MSZ participated in conception development, drafting and revising the manuscript, and giving final approval of the version to be published; AW participated in drafting the manuscript; HY participated in conception development and drafting the manuscript. All authors read and approved the final manuscript.
